# Current News

**Published:** 1903-02

**Authors:** 


### Current News.

### AMERICAN SOCIETY OF ORTHODONTISTS.

At the second annual meeting of the American Society of Orthodontists, held in Philadelphia, October 8, 9, and 10, 1902, the following officers were elected:

President, Milton T. Watson, D.D.S., Detroit, Mich.; Vice-President, Lloyd S. Lourie, D.D.S., Chicago, Ill.; Secretary, Anna Hopkins, D.D.S., St. Louis, Mo.

*Board of Censors.*—Richard Summa, D.D.S., St. Louis, Mo.; H. E. Lindas, D.D.S., Larned, Kan.; F. M. Casto, D.D.S., Columbus, Ohio.

*Committee on History and Invention.* — E. A. Bogue, M.D., D.D.S., New York, N. Y.; W. J. Brady, D.D.S., Iowa City, Iowa; Lloyd S. Lourie, D.D.S., Chicago, Ill.

Anna Hopkins,
*Secretary.*

St. Louis, Mo.

### ODONTOGRAPHIC SOCIETY OF CHICAGO.

At the annual meeting of the Odontographic Society of Chicago the following officers were elected for 1903:

President, F. B. Noyes; Vice-President, J. P. Buckley; Secretary, F. H. Zinn; Treasurer, G. N. West.

*Board of Directors.*—F. E. Roach, L. O. Green, H. A. Drake.

*Board of Censors.*—D. M. Cattell, W Girling, D. A. Hare.

The celebration of the fifteenth anniversary of this Society will be held on February 16 and 17, 1903. It will be a memorable meeting in many ways. Papers on live topics are already promised by representative men from all sections of the country. More than one hundred and fifty clinics will be given, on every operation of interest to dentists. The profession is cordially invited to come to Chicago and be entertained at that time. The meeting will not only be profitable, but pleasant.

Frank H. Zinn,
*Secretary.*

100 State Street, Chicago.

